# Chromosomal characterization of three species of Serrasalmini (Serrasalmidae: Characiformes)

**DOI:** 10.1590/1678-4685-GMB-2023-0088

**Published:** 2023-11-10

**Authors:** Alan Gomes dos Santos, José Francisco de Sousa e Souza, Simone Cardoso Soares, Celeste Mutuko Nakayama, Eliana Feldberg

**Affiliations:** 1 Instituto Nacional de Pesquisas da Amazônia (INPA), Programa de Pós-graduação em Genética, Conservação e Biologia Evolutiva, Laboratório de Genética Animal, Manaus, AM, Brazil.; 2 Instituto Nacional de Pesquisas da Amazônia (INPA), Coordenação de Biodiversidade, Laboratório de Genética Animal, Manaus, AM, Brazil.

**Keywords:** Piranha, chromosomal evolution, repetitive DNA, synteny

## Abstract

The tribe Serrasalmini is a diverse group with paraphyletic genera and taxonomic uncertainties. Several studies have been carried out in this group of fish in order to understand this problem, including the cytogenetic approach. In this study, three species of a clade of Serrasalmini were characterized cytogenetically - *Pristobrycon striolatus*, *Catoprion absconditus* and *Pygopristis denticulatus*. The three species presented diploid number (2n) equal to 62 chromosomes, of one and two arms, with karyotypic formulas and species-specific fundamental numbers. Heterochromatin is centromeric and terminal (bi-telomeric) in most chromosomes, with a conspicuous interstitial block at pair 1 (m) in all three species. The nucleolar organizer regions were multiple and C-band positive, and their location was confirmed via 18S ribosomal DNA mapping; however, with additional sites. The 5S rDNA was located in interstitial region of long arm of pair 1 (m), in the three species (homeologous). Moreover, we observed synteny between 18S and 5S in the species *C. absconditus* and *P. denticulatus*, which, according to fiber-FISH, are interspersed. Thus, the maintenance of 2n (62) evidences the diversification of chromosomal formulas within the clade by non-Robertsonian rearrangements and reflects the paraphyly of the related species.

## Introduction

Serrasalmidae constitutes a monophyletic group that has approximately 100 valid species, which are distributed in 16 genera (Fricke *et al*., 2023). They are popularly known as “pacus” and “piranhas”, and are endemic to the Neotropical region, where they inhabit a wide variety of water bodies, including the main channels of rivers, lakes, flooded forests, to environments of rapids, and have wide distribution and abundance in the Amazon, Orinoco and Paraná-Paraguay basins (Goulding, 1980; Machado-Allison, 1983; Jégu, 2003; Mateussi et al., 2020a ).

Several studies have shown that “piranhas” and “pacus” form a well-defined group within the order Characiformes, composing the family Serrasalmidae, which is divided into three clades (Ortí et al., 1996, 2008; Calcagnotto et al., 2005; Mateussi et al., 2020 a ; Kolmann et al., 2021). Based on analyses of mitochondrial and nuclear sequences, Thompson et al. (2014) corroborated the subdivision into three clades: the “pacu clade”, the “myleus clade” and the “piranha clade”, the former being considered basal and the latter as more derived. Mateussi *et al*. (2020a) proposed a phylogenomic hypothesis with ultra-conserved elements, in which all living genera of the family were included, which had a new intrafamilial classification with two subfamilies: Colossomatinae Kolmann *et al*. (2021) and Serrasalminae Bleeker 1859, the latter with two tribes: Myleini Eigenmann 1903 and Serrasalmini Bleeker 1859. The morphological characteristics for each subfamily involve the absence of a pre-dorsal spine in Colossomatinae and its presence in Serrasalminae, which is continuous to the first ray of the dorsal fin in the tribe Myleini and discontinuous in Serrasalmini.

Although this division is well defined, there are significant inter- and intraspecific variations within each clade, mainly with regard to allometry and coloration patterns, which are observed during their development or reproductive stage, as well as their morphology and distribution (Nico and Taphorn, 1988; Jégu, 2003; Queiroz et al., 2013). Within the tribe Serrasalmini (piranhas), for example, there are taxonomic uncertainties, with divergences in the relationship between the genera *Pristobrycon* and *Serrasalmus*. Machado-Allison (1985), in a morphological analysis, observed the non-monophyly of *Pristobrycon*, with species more related to *Serrasalmus*, and only *P. striolatus* closer to the genus *Pygopristis*. According to Ortí et al. (1996, 2008) and Thompson et al. (2014), *P. striolatus* is more closely related to *Catoprion* and *Pygopristis denticulatus*, and the other species of *Pristobrycon* (e.g., *Pristobrycon calmoni*) were grouped within *Serrasalmus*. The genus *Catoprion*, since its description in 1844 was considered monotypic (Fricke *et al*., 2023), and had a new species described: *C. absconditus* Mateussi, Melo & Oliveira, 2020, which occurs in the Amazon and Essequibo basins (Mateussi *et al*., 2020b).

Cytogenetic data in the family Serrasalmidae demonstrate that the fish of this family have high karyotypic diversity (Favarato et al., 2021). However, the clade formed by *Catoprion*, “*Pristobrycon*” and *Pygopristis* still lacks cytogenetic analysis, because, although 2n=62 has already been suggested by Nakayama (personal communication), no chromosomal data on these species are found in the literature. Thus, the objective of this study was to cytogenetically characterize *Catoprion absconditus* Mateussi, Melo & Oliveira, 2020, *Pristobrycon striolatus* (Steindachner, 1908) and *Pygopristis denticulatus* (Cuvier, 1819), which form a clade in the tribe Serrasalmini.

## Material and Methods

In the present study, three species of Serrasalmini were analyzed (Table 1). These suspensions were obtained from several collections by the researcher Dr. Celeste Nakayama (*in memoriam*), in the period from 1987 to 2009, under a permanent license (Nº 28095-3) granted by the Brazilian Institute of the Environment and Renewable Resources (IBAMA).

**Table 1 -:** Number of individuals analyzed in this study, according to species and sex.

Species	Individual		Location	Coordinates	Voucher
	Male	Female			
*Catoprion absconditus*	10	11	Uatumã River	1°54′56.7″ S, 59°28′25″ W	INPA-ICT 059837
*Pygopristis denticulatus*	2	4	Demini River	1°44’45” S, 62°93’31” W	INPA-ICT 059838
*Pristobrycon striolatus*	9	10	Anavilhanas	2°23’41” S, 60°55’14” W	INPA-ICT 059839

Chromosomal preparations were obtained from the kidney, according to the protocol of Bertollo et al. (1978), after mitotic induction with biological yeast (Oliveira et al., 1988). Colchicine 0.0125% was applied *in vivo* for 50 minutes. The detection of heterochromatin followed Sumner (1972), and the staining was according to Lui et al. (2012), which uses a solution of propidium iodide (0.5 µL propidium iodide in 20 µL Vectashield^®^ antifade). For the detection of nucleolar organizer regions (NORs), we used the silver crystal precipitation technique (Ag-NORs) as described by Howell and Black (1980).

Genomic DNA extraction was performed from the muscle tissue and liver of the species under study, which was preserved in 100% ethanol, using the Wizard^®^ extraction kit (Promega), following the manufacturer’s recommendations. The repetitive sequences, used as a probe, were isolated using PCR. The isolation of the 18S and 5S ribosomal genes was performed using the following primers: 18S: 18S*f* (5’-CCG CTT TGG TGA CTC TTG AT-3’) and 18S*r* (5’-CCG AGG ACC TCA CTA AAC CA-3’) (Gross et al., 2010), and 5S*f* (5’-TAC GCC CGA TCT CGT CCG ATC3’) and 5S*r* (5’- CAG GCT GGT ATG GCC GTA AGC 3’) (Martins and Galetti Jr, 1999). Double-stranded PCR products were obtained in a total volume of 25 µL (~100 ng of genomic DNA; 1x buffer; 0.5 unit of Taq DNA polymerase; 0.2 mM of each dNTP - dATP, dCTP, dTTP, dGTP; 0.2 µM of each oligonucleotide primer; 2.0 mM of magnesium chloride and Milli-Q water to complete the volume). The reactions were processed in thermocycler (Eppendorf Mastercycler Gradient). The PCR program was used with the following steps: 18S rDNA: 1 min at 95 °C (for denaturation of the DNA strand); 35 cycles of 1 min at 94 °C, 1 min at 56 °C (annealing) and 90 s at 72 °C (amplification) and 5 min at 72 °C (final extension). rDNA 5S: 1 min at 94 °C (denaturation); 35 cycles of 1 min at 94 °C, 1 min at 55 °C (annealing) and 90 s at 72 °C (amplification) and 5 min at 72 °C (final extension). After amplification, the PCR products were verified and quantified. For the telomeric probes, the primers (TTAGGG)5 and (CCCTAA)5 were used according to Ijdo et al. (1991). The PCR products, 18S ribosomal DNA (rDNA) and the telomeric sequences were labeled using the Atto Nick Translation labeling kit (Jena Bioscience) method 550 - red and the rDNA 5S, 488 - green, following the manufacturer’s instructions. The fluorescence *in situ* hybridization (FISH) technique was as per described by Pinkel et al. (1986) and the Fiber-FISH technique was performed according to Barros et al. (2011). The slides that used fluorochromes (C-banding and FISH) were analyzed under an epifluorescence photomicroscope (Olympus, BX-51) using an appropriate filter. At least 30 metaphases per individual were analyzed, and the best had their image captured using the DPController image capture system and were processed using the DPManager program. To assemble the karyotypes, we used Adobe Photoshop 7.0 (version CS6), by which mitotic metaphase chromosomes were cut, paired, measured in the DPManager program, and placed in descending order of size. The morphology and classification of the chromosomes were determined according to Levan et al. (1964) and, to determine the number of arms (FN), the metacentric (m), submetacentric (sm) and subtelocentric (st) chromosomes were considered as having two arms and the acrocentric (a) as having only one arm.

## Results

**Figure 1 -:**
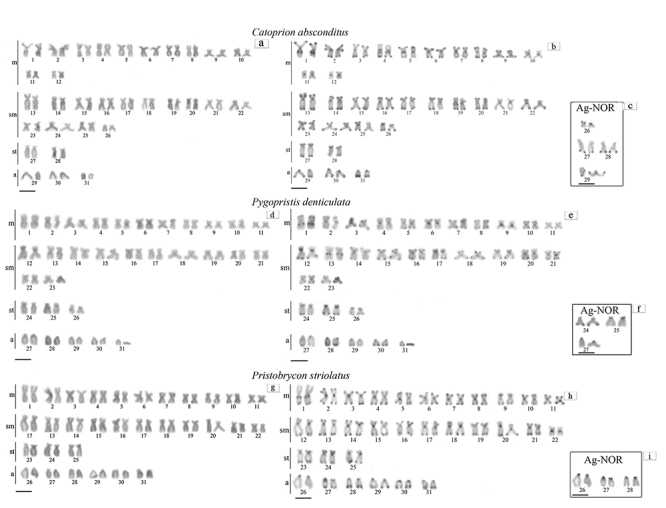
Karyotypes analyzed in conventional Giemsa stain, C banding and nucleolar organizer regions (NOR, box) of *C. absconditus* (a, b, c), *P. denticulata* (d, e, f) and *P. striolatus* (g, h, i). Scale bar=5μm.

The three species presented the diploid number (2n) equal to 62 chromosomes, and *Catoprion absconditus* has an FN=118 and KF=24m+28sm+4st+6a; *Pygopristis denticulatus* has FN=114 and KF=22m+26sm+4st+10a; *Pristobrycon striolatus* has FN=112 and KF=22m+22sm+6st+12a (Figure 1a, d, g). No heteromorphic sex-chromosome was found.

The heterochromatin (HC) of the three species is located, preferably, in the centromeric and terminal (bi-telomeric) regions of the chromosomes. However, some blocks are species-specific, especially those that are interstitial (Figure 1b, e, h).

In *Catoprion absconditus*, interstitial heterochromatic markings were evidenced in the long arms of pairs 1, 4, 5 (m), 19 (sm), in the short arms of pair 13 (m), and were only centromeric in pairs 10, 11 and 12 (m) and 21 (sm) (Figure 1b). In *Pygopristis denticulatus*, interstitial markings were evidenced in the long arms of pairs 1 (m), 13 (sm) and were only centromeric in pairs 4, 9 and 11 (m). Pairs 7 and 8 (m) showed bi-telomeric markings, and pairs 6 (m) and 20 (sm) showed centromeric and telomeric markings in only one arm (Figure 1e). On the other hand, in *Pristobrycon striolatus*, interstitial blocks appeared only in pair 1 (m), and pair 22 (sm) showed only centromeric marking and pair 27 showed centromeric and terminal marking in the long arm, while the other pairs have terminal marking, which is sometimes bi-telomeric (Figure 1h).

**Figure 2 -:**
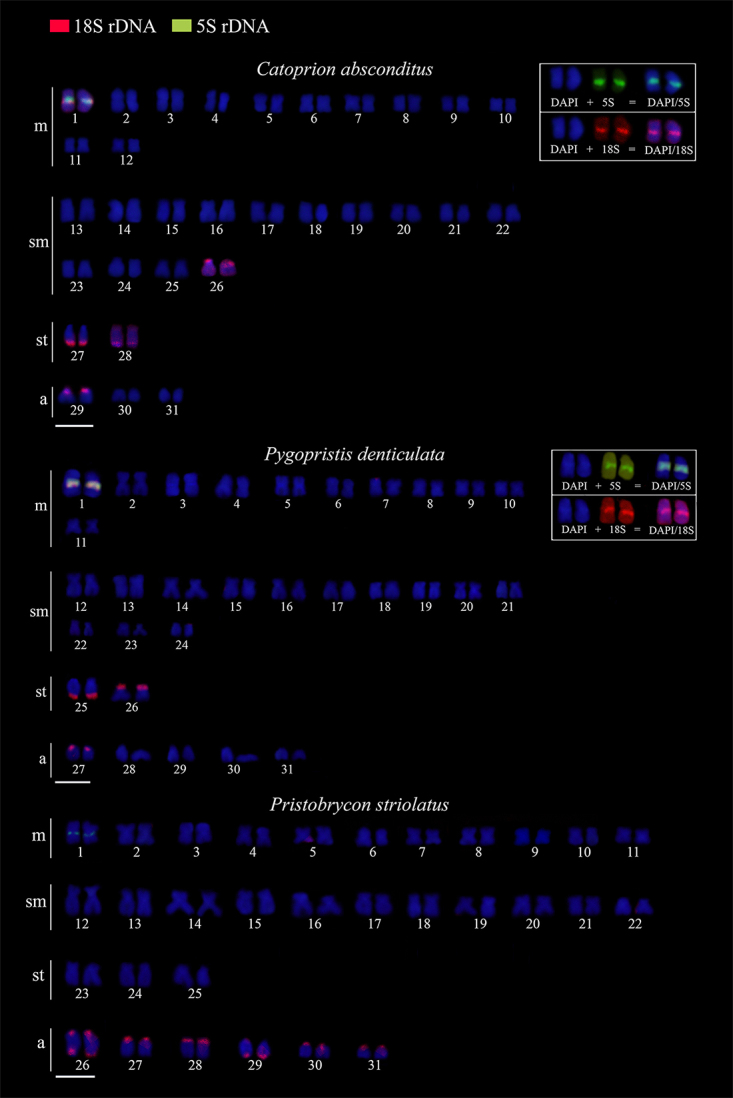
Chromosomal mapping of 18S (red) and 5S (green) rDNA. Scale bar: 5μm.

**Figure 3 -:**
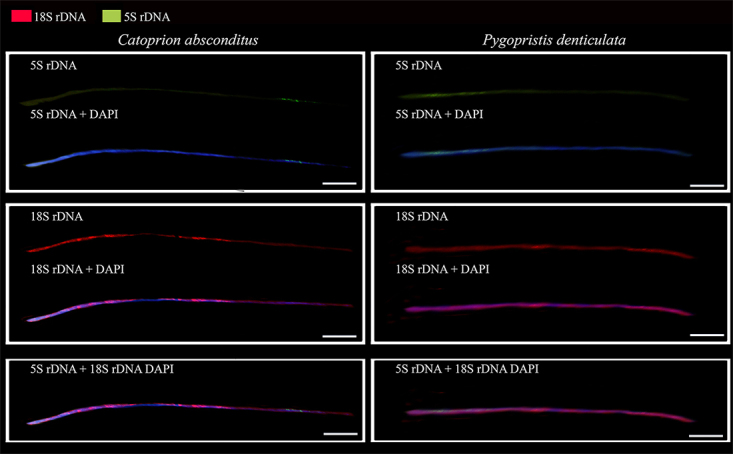
Fiber-FISH with 18S (red) and 5S (green) rDNA, DAPI (blue). Scale bar: 5µm.

The Ag-NORs were multiple and were evidenced in 3 to 4 chromosomal pairs. They were all C-band positive and located in the terminal portions of the chromosomes (Figure 1c, f, i). The ribosomal DNA mapping of 18S confirmed the location of the active Ag-NORs in the three species analyzed. However, additional sites were evidenced and, in *C. absconditus* and *P. denticulatus*, this additional site is in an interstitial position in pair 1 (m), colocalized with the heterochromatin block (C^+^) and in synteny with 5S rDNA (Figure 2). In *P. striolatus*, we found 14 sites on acrocentric chromosomes, six coinciding with the Ag-NORs (pairs 26, 27, 28) and additional terminal markings on pairs 26*q*, 29*q*, 30*p* and 31*p*. The mapping of 5S rDNA showed only one marked pair in the three species, in an interstitial position in pair 1 (m) (Figure 2). By means of the Fiber-FISH technique, the extended fibers showed that the colocalized ribosomal genes in *C. absconditus* and *P. denticulatus* are adjacent and have the variable presence of the ribosomal DNA classes (Figure 3).

**Figure 4 -:**
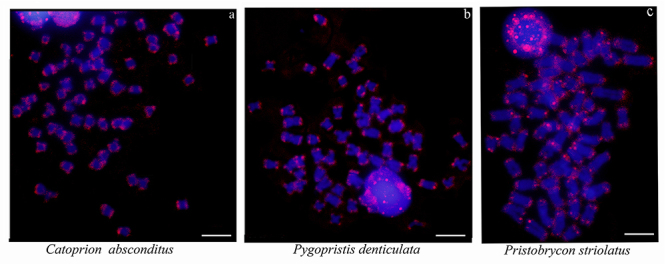
Probes with telomere sequences (TTAGGG)_n_ (red) and DAPI (blue). Scale bar: 5μm.

Telomeric sequences (TTAGGG)_n_ were evidenced in the terminal portions of all chromosomes of the three species (Figure 4), and interstitial telomeric sequences (ITS) were not evidenced.

**Figure 5 -:**
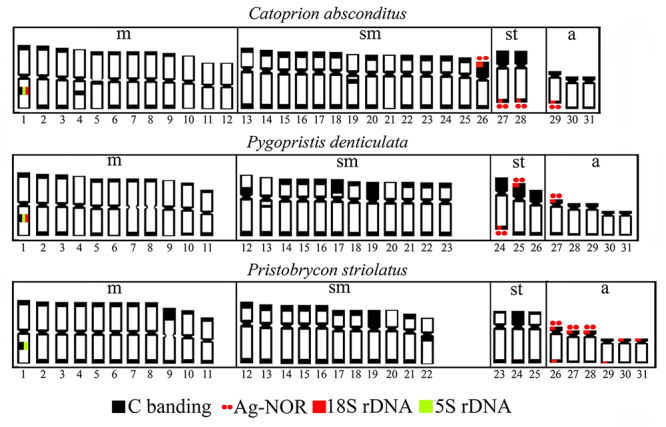
Schematic representation of the chromosomes of the three species, compiling the C-band, Ag-NOR, 18S and 5S rDNA data.

In Figure 5, we highlight the cytogenetic characteristics of the three species analyzed here, which included 2n=62, the interstitial heterochromatin block of pair 1 (m), the location of the 5S rDNA (pair 1) and the syntheny of the heterochromatic block with 5S and 18S rDNA, but not with Ag-NOR in the species *C. absconditus* and *P. denticulatus*.

## Discussion

In the family Serrasalmidae, the diploid number varies from 54 to 64; however, within each clade, 2n seems to be conserved (Favarato et al., 2021), except in *Serrasalmus* (Muramoto et al., 1968; Nakayama et al., 2002). Our data showed 2n=62 chromosomes in the three species analyzed, with 2n being shared with species of the genus *Metynnis* (Favarato *et al*., 2019, 2021). Nonetheless, although 2n is conserved among these species, the karyotypic formulas (KF) differ from each other, mainly by the number of st-a chromosomes, thus evidencing the presence of non-Robertsonian rearrangements such as inversions, translocations, duplications and heterochromatinization, given the presence of chromosomes with totally heterochromatic short arms (Figure 5).

In addition, it is curious to note the paraphyly in *Pristobrycon*, which is supported by morphological and molecular data (Machado-Allison, 1985; Mateussi et al., 2020a; Kolmann et al., 2021), which is also reflected by the cytogenetic data, since *P. striolatus* shares the diploid number with *Catoprion* and *Pygopristis* and with the only pacu of the tribe, *Metynnis*; while *Pristobrycon calmoni* (type species of the genus) shares the 2n with the “true piranhas”, such as *Serrasalmus* and *Pygocentrus* (2n=60) (Nakayama et al., 2008; Santana et al., 2011).

The location of heterochromatin, preferably in the centromeric and terminal regions (bi-telomeric), that is evidenced in the analyzed species, is common among Characiformes, and is associated with the preservation of some genes or even their silencing (Martins and Galetti Jr, 2001; Cioffi and Bertollo, 2012). In this sense, the constitutive heterochromatin blocks, here associated with the 18S and 5S rDNA sites, may be acting in the preservation of these genes or even acting in the silencing of pseudogenes associated with these sequences.

Fully heterochromatic short arms, C^+^ NORs, and 18S and 5S rDNA sites coincident with C^+^ blocks are common among Serrasalmidae, though species-specific interstitial blocks are also present (Nakayama et al., 2008, 2012; Ribeiro et al., 2014; Favarato et al., 2021). Considering the Serrasalmidae family, an increase in the diploid number and different karyotypic formulas are observed, which we associate with the presence of Robertsonian and non-Robertsonian rearrangements, but with a large participation of heterochromatin and probable mobile elements. Thus, we also observed several markers that can assist in the cytotaxonomy of the group.

A noteworthy fact is the conspicuous heterochromatic block that occurs in the interstitial position of pair 1 in the three species and is co-located with the 5S rDNA site, which has been considered a cytotaxonomic marker among Serrasalminae, since, in the genus *Serrasalmus*, this pair is in 7 (m) and in *Pygocentrus* species, this block is in pair 3 (m) (Cestari and Galetti Jr, 1992; Nakayama et al., 2001; Centofante et al., 2002 Nakayama *et al*., 2008, 2012). In this sense, we can infer that the pair carrying the interstitial heterochromatic block is homeologous among the species of the tribe Serrasalmini, and that non-Robertsonian rearrangements would have changed its position in the karyotype and, therefore, promoted diversification of the heterochromatin pattern in the different species of this tribe.

The presence of fully heterochromatic short arms in submetacentric and subtelocentric chromosomes was also observed in other representatives of the family, such as in the species *Serrasalmus* (Nakayama et al., 2002) and *Myloplus* (Favarato et al., 2021). This characteristic must have arisen due to *in tandem* duplication, especially in association with repetitive DNA elements, since the repetitive nature of these elements seems to be the trigger and target for heterochromatinization (Guimarães et al., 2016; Pinheiro et al., 2016).

The presence of chromosomal rearrangements in the evolution of the clade under study is also observed through the location of the nucleolar organizer regions (Ag-NOR and 18S rDNA), which presents wide interspecific variation. In *P. striolatus*, which has six acrocentric pairs, Ag-NOR is present in three of them, and 18S rDNA confirmed these sites; however, eight more 18S rDNA sites were detected, being a bi-telomeric pair, totaling 14 sites. In *P. denticulatus*, the Ag-NORs is present in one acrocentric pair, and in two subtelocentric pairs, with the 18S rDNA confirming the six sites of Ag-NORs but marking one more pair (1m). In *C. absconditus*, which has only three acrocentric pairs, Ag-NORs is present in one of them, and the other markings are in st and sm, with the 18S rDNA confirming these markings, but marking one more pair (1m).

The occurrence of additional 18S rDNA sites to those marked by silver nitrate, as seen in the species analyzed here, may be due to NORs dispersion events, which, due to their location in the terminal portion, are more prone to breakage, with consequent chromosomal rearrangements (Moreira-Filho et al., 1984; Pinheiro et al., 2016) or pseudogenes, by non-Robertsonian rearrangements of the translocation type or movements associated with heterochromatin or transposable elements (TEs) (Vicari et al., 2008; Terencio et al., 2012, 2015; Guimarães et al., 2016). 

In the genera of the basal clade of Serrasalmidae (2n=54), we found up to four sites of 18S rDNA, which, with the increase of the diploid number in the derived clades, caused the number of ribosomal sites to also increase. However, this is not true for all genera since in *Metynnis*, although its species also have 2n=62, the 18S rDNA sites are present on one or two pairs of chromosomes (Favarato et al., 2021). Notably, in this clade, there is an increase in the number of such sites, which reaches 14.

In the clade under study, NOR dispersal may have occurred by inversion or translocation, which are rearrangements that are closely related to ribosomal DNA families. Therefore, we suggest two scenarios: 1) pericentric inversion in nucleolar acrocentric chromosomes, which changes the morphology to two-arm chromosomes (subtelocentric/submetacentric); or 2) translocation of 18S rDNA sequences from an acrocentric to an interstitial position in metacentric chromosomes (pair 1). The first scenario is assumed due to the morphology of the chromosomes (with two arms) that maintain transcriptional activity (Ag-NOR). While in the second scenario, which did not present transcriptional activity, it would have been transposition or translocation facilitated by transposable elements (TEs), which preferentially invade rDNA regions with consequent diversity in their location within the karyotypes, due to their replicative nature (Biémont and Vieira, 2006). In *P. striolatus*, pair 26 (a) has bi-telomeric 18S rDNA and, in the region of the short arm, it is associated with HC; however, in the terminal region of the long arms, there is no HC. These sequences may be associated with TE (Terencio et al., 2015) and usually become inactive and prone to accumulation of mutations (insertions, deletions) at neutral rates until they completely lose their identity or become lost in the genome (Fernández-Medina et al., 2012).

The three species present the 5S rDNA in only one chromosome pair (pair 1), in an interstitial position, and its location in only one chromosome pair is considered an ancestral condition and may confer some advantage for protection of this gene in the genome of the species (Martins and Galetti Jr, 2000, 2001; Cioffi and Bertollo, 2012); however, several differences in number and location of this sequence (5S rDNA) were observed in the family Serrasalmidae, ranging from one to two pairs, with a terminal or interstitial position, but always colocalized with heterochromatin (Nakayama et al., 2008, 2012; Ribeiro et al., 2014; Favarato et al., 2019, 2021).

The clade that first diverged, Colossomatinae, presents the 5S rDNA sites located in two chromosomal pairs, in an interstitial position; while, in Serrasaminae, these sites are present in one or two pairs and, in species with one pair, the marking is always interstitial and, when it marks two pairs, in one, the marking is interstitial and, in the other, it is terminal (Nakayama et al., 2008, 2012; Ribeiro et al., 2014, Favarato et al., 2019, 2021). This indicates that non-Robertsonian rearrangements may have decreased the number of sites or that the marking in the second pair may be a pseudogene (Martins et al., 2002). On the other hand, we believe that the chromosomal pair that carries the 5S rDNA sequence in the interstitial position may be homeologous within the family Serrasalmidae (Nakayama *et al*., 2008, 2012; Ribeiro *et al*., 2014; Favarato *et al.*, 2019). In this sense, the great similarity between these chromosomal pairs may represent an important cytotaxonomic marker, since it allows the differentiation between species, such as those of the genera *Serrasalmus* and *Pygocentrus*, which are often confused because of their morphological similarity.

Another fact that deserves to be highlighted is the syntenic location of the 18S and 5S rDNA in *C. absconditus* and *P. denticulatus* species, for which the Fiber-FISH analysis showed the intercalated location of these rDNA sites (Figure 3). The location of the rDNA sequences on different chromosomes decreases the chances of disadvantageous rearrangements occurring during cell division (Martins and Wasko, 2004); however, in some species, the syntenic location of these rDNA sequences occur without observable damage to the host (Guimarães et al., 2016; Favarato et al., 2021; Souza et al., 2021; Moraes et al., 2022).

In Serrasalmidae, for example, the synteny between 18S and 5S rDNA was evidenced in three species of *Metynnis* and may confer an adaptive advantage for the maintenance of this organization (Favarato et al., 2021). In *Ctenolucius hujeta*, the co-location of the 18S and 5S rDNA sites was proposed as a reflection of a probable adaptive condition in the organization of these multigene families in the genome of these species (Souza et al., 2021). On the other hand, in Erythrinidae, in the species *Hoplias malabaricus* (Bloch, 1794), synteny may have been caused by the accumulation of repetitive DNA sequences, since the transposable elements of *Rex* 3 were mapped concomitantly with ribosomal genes (Guimarães et al., 2016).

The colocalization of these classes of rDNA is an unusual feature, since the dispersion of these multigene families is the result of independent events and can be associated with the silencing of these genes (Vicari et al., 2008; Barros et al., 2011; Sochorová et al., 2017). At this point, it is worth mentioning that, in both the species analyzed here, the 18S ribosomal DNA sites in synteny with 5S did not present transcriptional activity (using the Ag-NOR technique), which indicates that this site was inactive during the last interphase. Therefore, it is necessary to use other molecular markers, such as sequencing and characterization of gene family structures, since they could provide information about the function or pseudogenization, and cytogenetic markers, such as TE and histones, in order to better understand these events of synteny, dispersion, association and cytotaxonomic contributions (Traldi et al., 2019; Haerter et al., 2022).

Telomeric sequences (TTAGGG)_n_ were detected only in the terminal portions of all the chromosomes of the three species analyzed. It is interesting to note that, although the presence of rearrangements in Serrasalmidae is notorious, so far only one species analyzed presented interstitial telomeric sequences (ITS), i.e., *Colossoma macropomum* (which makes up the basal clade of the family), in a metacentric pair, and in a centromeric position, coinciding with heterochromatin (Ribeiro et al., 2014).

The cytogenetic characterization of these Serrasalmini species evidenced a cytotaxonomic marker, an interstitial heterochromatin block at pair 1 (m) associated with 5S rDNA. The diversification of karyotype formulae within the clade is also observed by the nucleolar organizer regions (Ag-NOR) and 18S rDNA, which corroborates the presence of non-Robertsonian rearrangements. The 2n=62 reinforces the maintenance of the diploid number and reflects their relationship as a sister group of *Metynnis*, this being a plesiomorphic condition in the tribe. In addition, the relationship with the *Pygocentrus* + *Serrasalmus* clade indicates the occurrence of Robertsonian chromosome rearrangements that led to 2n=60. It is noteworthy that with *Pristobrycon,* considered a junior synonym of *Serrasalmus*, the results found provide further evidence (cytogenetics) that *Pristobrycon striolatus* needs a new genus in which to allocate this species.
